# Single‐nuclei RNA‐Sequencing of Postmortem Choroid Plexus Uncovers Dysfunction Alzheimer’s disease

**DOI:** 10.1002/alz.094997

**Published:** 2025-01-09

**Authors:** Tristan J Philippe, Nicola Kearns, Denis R. Avey, Devin M. Saunders, Himanshu Vyas, Sashini L De Tissera, Jishu Xu, David A. Bennett, Yanling Wang

**Affiliations:** ^1^ Rush Alzheimer’s Disease Center, Rush University Medical Center, Chicago, IL USA; ^2^ Department of Neurological Sciences, Rush University Medical Center, Chicago, IL USA

## Abstract

**Background:**

The choroid plexus (ChP) is formed by epithelial cells and stromal fibroblasts which act as a blood‐cerebrospinal fluid (CSF) barrier, play a key role in maintaining brain homeostasis, and provide a niche for immune cells. ChP dysfunction has been implicated in Alzheimer’s Disease (AD), including changes in CSF secretion, increased apoptosis, and dysregulated immune, mitochondrial, and transporter functions.

**Method:**

Here, we performed single‐nuclei RNA‐Sequencing (snRNA‐Seq) on 965,647 ChP nuclei from 68 ROSMAP participants with no cognitive impairment (NCI), mild cognitive impairment (MCI) or Alzheimer’s Dementia (ADem).

**Result:**

We identified 14 major cell types, including two types of epithelial cells, four types of fibroblasts, five types of border associated macrophages (BAMs), one type of T cells, and two types of vascular cells. In ciliated epithelial cells, we observed a decreased expression of genes essential for cilia formation and movement (e.g., DNAH12, CFAP100, ARL13B) in participants with ADem, but not MCI. In ADem and MCI fibroblasts and epithelial cells, we observed changes in cell adhesion (e.g., ITGA8, CEMIP, CEACAM1, ADMTS4), ion transport (e.g., SLC12A2, SLC4A5, STAEP4, SLC1A1), and metabolism (e.g., ALDH4A1, ENO4, AKR1C2, PDE10A) in addition to increased expression of pro‐inflammatory genes (e.g., IL6, CXCL8, IL12RB2). Compared to the NCI group, we also observed increased expression of lipid metabolism genes (e.g., ALOX15B, ACSL1) in phagocytic BAMs and a higher proportion of pro‐inflammatory BAMs in the ADem, but not MCI groups. Proteomics on ex‐vivo CSF and tissue, immunostaining, and RNAscope support the major cell types described herein and validate differential gene expression.

**Conclusion:**

We show that multiple cellular and molecular functions are dysregulated in ADem, to suggest ChP dysfunction is a key contributor to AD pathogenesis. Changes to the cilia of ChP epithelial cells and ChP BAM function could contribute to differences in the degree of cognitive impairment across participants.

